# Serrated polyposis syndrome and colonoscopic surveillance: who is it safe to follow?

**DOI:** 10.1186/1897-4287-10-S2-A20

**Published:** 2012-04-12

**Authors:** S Parry, S Woodall, G Willdridge, MD Walsh, DD Buchanan, C Rosty, JP Young

**Affiliations:** 1 New Zealand Familial Gastrointestinal Cancer Registry, Auckland Hospital, New Zealand; 2 Department of Gastroenterology, Middlemore Hospital, Auckland, New Zealand; 3 Familial Cancer Laboratory, QIMR, Herston, Queensland, Australia; 4 Department of Molecular and Cellular Pathology, University of Queensland, Queensland, Australia; 5 School of Medicine, University of Queensland, Herston, Queensland, Australia

## Aim

To assess colorectal cancer (CRC) risk during colonoscopy surveillance in a cohort of patients with the serrated polyposis syndrome (SPS).

## Method

Colonoscopy and histology records from January 2000 to time of interview for 67 New Zealand patients, meeting WHO criteria for a diagnosis of SPS and enrolled in the Genetics of Serrated Neoplasia study, were reviewed. Polyp demographics, smoking status and family history of cancer were recorded.

## Results

Of 67 patients 18 presented with CRC (mean age 57yrs, 12 Female). Only one of 18 reported a first degree relative (FDR) with CRC. Over a median follow-up of 8 years from time of surgery with an average interval of 16 months between colonoscopies, two patients developed metachronous CRC: one identified at prophylactic completion colectomy and one in association with first diagnosis of SPS 19 yrs following initial CRC and 30 months after a previous colonoscopy. Of 45 patients with polyps alone (mean age 48 yrs, 27 female) followed for a median of 9 years with an average interval of 15 months between colonoscopies, none developed CRC despite 33 having multiple pan colonic polyps. Of these 33, 70% had an adenoma, 18 % a sessile serrated polyp and 53 % polyps >10 mm. A FDR with CRC was reported in 14/45 (31%). Four patients with multiple pan colonic polyps and no FDR with CRC underwent prophylactic subtotal colectomy (mean age 58 yrs, 3 females) after being followed for a median of 5 years with an average interval between colonoscopies of 8 months. All had adenomas and one a sessile serrated polyp. No significant smoking effect was seen in any group.

## Conclusion

If endoscopic control is feasible, SPS patients can be judiciously managed by frequent surveillance colonoscopy.

